# Choroidal bulging in patients with Vogt-Koyanagi-Harada disease in the non-acute uveitic stage

**DOI:** 10.1186/1869-5760-4-6

**Published:** 2014-02-18

**Authors:** Viviane M Sakata, Felipe T da Silva, Carlos E Hirata, Walter Y Takahashi, Rogerio A Costa, Joyce H Yamamoto

**Affiliations:** 1 Department of Ophthalmology, Faculdade de Medicina, Universidade de São Paulo, São Paulo SP 05403-000, Brazil; 2 Division of Macula: Imaging & Treatment, Centro Brasileiro de Ciências Visuais, Belo Horizonte MG 30150-270, Brazil; 3 Department of Ophthalmology, Faculdade de Medicina de Ribeirão Preto, Universidade de São Paulo, Ribeirão Preto SP 14048-900, Brazil; 4 Rua Marechal Hermes, 872. Centro Cívico, Curitiba PR 80.530-230, Brazil

**Keywords:** Vogt-Koyanagi-Harada disease, Spectral-domain optical coherence tomography, Non-acute uveitic stage, Disease activity parameter, Choroidal inflammation

## Abstract

**Background:**

Detection of choroidal inflammation in Vogt-Koyanagi-Harada (VKH) disease is still a challenge. Progression to sunset glow fundus has been observed despite apparent good clinical control of inflammation. Indocyanine green angiography (ICGA) permits choroid inflammation detection, though it is invasive, time consuming, and costly. The purpose of the present study is to report a sign indicative of probable inflammation on enhanced depth imaging spectral-domain optical coherence tomography (EDI-OCT): a localized increase in choroidal thickness with bulging of the outer retina (‘choroidal bulging’) in patients with VKH disease in the non-acute uveitic stage.

**Findings:**

This is a retrospective observational study. The choroidal bulging was a particular finding observed in four eyes of three patients with VKH disease in the non-acute uveitic stage (median disease duration 55.3 ± 40.3 months, range 10 to 108). This study is part of an ongoing longitudinal study in patients with VKH disease carried out in the Uveitis Service, Hospital das Clínicas, Faculdade de Medicina, Universidade de São Paulo, São Paulo, Brazil. In all eyes, the choroidal bulging was identified in the presence of anterior chamber cells and/or on fundus angiographic (fluorescein and indocyanine green) findings, indicative of disease activity. Changes in the thickness of the choroidal bulging accompanied the variation in the clinical and angiographic signs of inflammation.

**Conclusion:**

The choroidal bulging is a particular finding detected on EDI-OCT that may indicate ongoing inflammation in the posterior segment of the eye. This EDI-OCT feature may assist in the treatment-monitoring of patients with Vogt-Koyanagi-Harada disease in the non-acute uveitic stage.

## Findings

### Background

Vogt-Koyanagi-Harada (VKH) disease is an autoimmune disorder, in which the choroid is the main site of disease-related inflammation in the eye [1]. Detection of choroidal inflammation is still a challenge, and progression of VKH disease has been observed despite apparent good clinical control of inflammation. Indocyanine green angiography (ICGA) has been used as a sensitive technique for imaging choroidal inflammation. However, it is limited by the fact that it is invasive, time consuming, and costly. Moreover, recent reports have risen up that some changes do not represent active lesions but intrastromal scars [2].

Newer imaging techniques, such as enhanced depth imaging optical coherence tomography (EDI-OCT), have been recently evaluated as an alternative modality of monitoring treatment effect. In this context, we describe a unique finding in patients with VKH disease in the non-acute uveitic stage: the ‘choroidal bulging’, which seems to be related to active inflammation. This finding appears as a localized thickening of the choroid that assumes a convex appearance with consequent bulging of the adjacent retina, with no nearby obvious retinal lesion to justify this finding. Although diffuse thickening of the choroid has already been described in some retinal diseases (including VKH disease), we are not aware of other conditions that commonly produce choroidal bulging (i.e., a *localized* thickening of the choroid) [3,4].

## Methods

We retrospectively reviewed sequential clinical and imaging data from all eyes noted to have the choroidal bulging on EDI-OCT performed as part of the follow-up protocol in an ongoing longitudinal VKH disease study, which is being conducted at the Uveitis Section, Hospital das Clínicas, Faculdade de Medicina, Universidade de São Paulo, São Paulo, Brazil. The study protocol followed the statements of the Declaration of Helsinki and was approved by the Universidade de São Paulo Institutional Review Board. In accordance with the Revised Diagnostic Criteria for VKH disease [5], incomplete VKH disease was diagnosed in all cases. The patients included were in the non-acute uveitic stage, with median disease duration of 55.3 ± 40.3 months (range 10 to 108 months). Fundus photography was performed using a conventional fundus camera system (TRC-50X/IMAGEnet, Topcon Inc., Tokyo, Japan). Fluorescein angiography (FA), ICGA, and EDI-OCT imaging were performed using Spectralis® HRA + OCT (Heidelberg Engineering Inc., Heidelberg, Germany).

## Results

One area of localized thickening of the choroid that assumes a convex appearance with consequent bulging of the adjacent retina (i.e., choroidal bulging) was identified in at least one follow-up visit in three patients. In all cases, at least two sequential visits were analyzed. Observation of the same scan area on EDI-OCT revealed appearance/disappearance of the choroidal bulging during the period analyzed. One patient had bilateral involvement and will be described further. At the time of identification of the choroidal bulging, all four eyes presented at least one concomitant inflammatory sign (anterior chamber cells on clinical examination and/or optic disc hyperfluorescence on FA and/or dark dots on ICGA). The changes observed in the thickness of the choroidal bulging along with the sequential follow-up EDI-OCT evaluations coincided with the variation in these inflammatory signs (Table 1).

**Table 1:** Characteristics of patients with choroidal bulging and Vogt-Koyanagi-Harada disease in non-acute uveitic stage

**Patient, gender, age (years)**	**Studied eye**	**Disease duration (months)**	**Choroidal bulging**		**Signs of disease activity**				**Changes observed at subsequent FU visit**
			**Location**	**Change**	**ACC**	**Hyperfluorescence of OD on FA**	**Dark dots on ICGA**	**Medication adjustment after CB visit**	
1, M, 65	RE	117	Outer nasal	+139 μm (+74%)	+/2	−	+	None	Month 137: improvement of CB (−145 μm), increase in ACC (+), absence of dark dots (−)
2, F, 54	RE	10	Outer nasal	+131 μm (+139%)	+	++	+++	Tapering of oral prednisone (15 to 10 mg/day)	Month 17: worsening of CB (+66 μm), increase in OD hyperfluorescence (+++), localized increase of dark dots
	LE	10	Outer nasal	+138 μm (+107%)	+	++	+++		Month 17: improvement of CB (−101 μm), decrease in OD hyperfluorescence (+/2), decrease of dark dots (+)
3, F, 53	LE	32	Parafoveal	+215 μm (+74%)	+	+	++	Substitution for mycophenolate mofetil 1.5 g/day	Month 35: disappearance of CB (−74 μm), maintenance in OD hyperfluorescence (+), attenuation of dark dots

These characteristics were observed by the time the choroidal bulging was identified on enhanced-depth imaging spectral optical coherence tomography. ACC, anterior chamber cells; CB, choroidal bulging; F, female; FA, fluorescein angiography; FU, follow-up; ICGA, indocyanine green angiography; LE, left eye; M, male; OD, optic disc; RE, right eye.

## Case report

**Figure 1:**
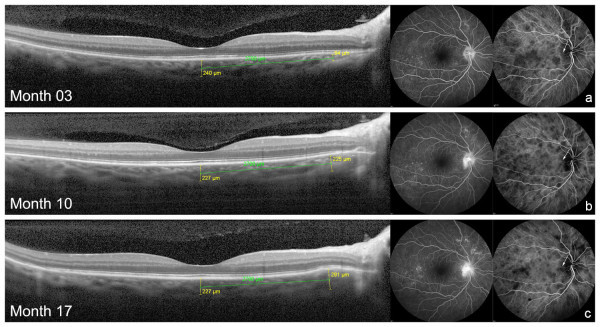
**Choroidal bulging and angiographic findings in the RE of case 2.** Absence of abnormalities on EDI-OCT at 3 months of disease in RE **(a)**. Gradual increase in choroidal thickness (choroidal bulging) in the outer nasal macular sub-field at months 10 **(b)** and 17 **(c)** was associated with gradual increment in optic disc hyperfluorescence on FA and localized appearance of dark dots on ICGA (arrows). Fluctuation in the diffuse density of dark dots on ICGA was concomitantly observed.

**Figure 2:**
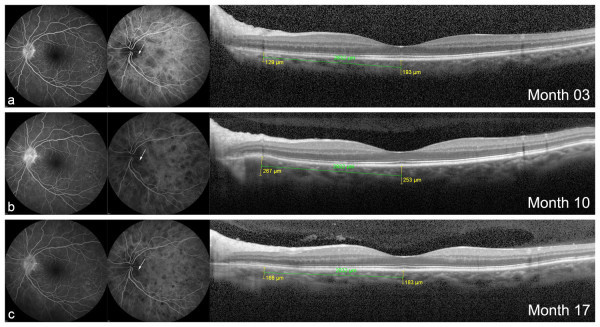
**Choroidal bulging and angiographic findings in the LE of case 2. (a)** Absence of abnormalities on EDI-OCT at 3 months of disease in LE. Focal changes (increase and decrease) in choroidal thickness in the outer nasal macular sub-field at months **(b)** 10 and **(c)** 17, in agreement with the changes observed in the optic disc hyperfluorescence on FA as well as in the localized (arrows) dark dots on ICGA.

A 54-year-old woman with VKH disease diagnosed 3 months ago came for routine evaluation (*Patient 2*, Table 1). She was under 40 mg of oral prednisone. Best-corrected visual acuity was 1.0 in OU; there were no cells in the anterior chamber in OU. Fundus examination showed a moderate diffuse depigmentation in OU (mild fundus) [6]. Angiographic signs of ongoing disease activity were observed, such as diffuse staining of the optic disc on FA and numerous, large and coalescent dark dots at the posterior pole and slightly contiguous to the temporal rim of the disc on ICGA in both eyes. Subfoveal choroidal thickness on EDI-OCT was around normal ranges (240 μm in right eye (RE) and 193 μm in left eye (LE)) and no choroidal folds or bulging was observed at this time (Figures 1a and 2a). Seven months later, EDI-OCT scan demonstrated a localized thickening of the choroid in the papillomacular bundle in OU (+131 μm in RE and +138 μm in LE) with a discrete but evident bulging of the outer retina (Figures 1b and 2b). Biomicroscopy revealed the presence of anterior chamber cells (1+ RE and traces LE). At this time, the patient was in use of prednisone 15 mg. Topical steroids were started, and prednisone dose taper was maintained as part of a dose reduction protocol. At her next visit, 7 months later, choroidal bulging was indeed noticed with an additional localized increase of 66 μm in choroidal thickening in the RE and diminishment in the LE (−101 μm) (Figures 1c and 2c). FA and ICGA changes followed the increase in the choroidal bulging in the RE: on FA, optic disc leakage became evident and more intense, and on ICGA, two dark dots could be clearly delineated at the exact place of the choroidal bulging (Figure 1c, arrows). In the LE, optic disc leakage became less intense on FA, and localized dark dots persisted on ICGA at choroidal bulging location, though more attenuated (Figure 2c, arrows). Traces of cells in the anterior chamber were still observed in OU. Oral prednisone was maintained at 10 mg/day.

## Discussion

In VKH disease, detection of choroiditis is still a challenge. Thus, newer parameters to detect posterior segment inflammation and to assist the treatment process are needed. Herein, we describe the choroidal bulging, a transient finding identified on EDI-OCT in patients with VKH disease in the non-acute uveitic stage. The choroidal bulging is characterized by a localized thickening of the choroidal compartment with consequent bulging of the RPE/Bruch reflective complex anteriorly, without an associated retinal thickening or any obvious nearby retinal lesion to justify this finding. Signs of well-known ongoing disease-related inflammation/activity were concomitantly observed in all four eyes presenting choroidal bulging, and fluctuation of this inflammatory signs was followed by changes in the choroidal bulging.

Diffuse thickening of the choroid has already been described in the early and convalescent/chronic stages of VKH [3,4], and it was suggested to represent a sign of disease activity [3,4]. Nevertheless, as previously described, progressive choroidal thinning related to disease duration occurs, and diffuse thickening during disease exacerbation in the non-acute uveitic stage of the disease may be subtle and difficult to detect [4,7]. Thus, the localized thickening of the choroid, the choroidal bulging, may represent a new sign indicative of posterior segment inflammation in a non-invasive manner.

Localized choroidal thickening has already been described in patients with ocular toxoplasmosis or choroidal tumors [8,9]; however, different from the choroidal bulging observed in this series of patients with VKH disease, an associated retinal or choroidal lesion was invariably observed in these scenarios on clinical exam (i.e., fundoscopy).

Choroidal bulging could be correlated to choroidal folds, despite the fact that these are multiple and described at the acute phase of the disease [10]. Choroidal folds or striations have been described as undulations and bumps on the surface of the retinal pigment epithelium [10]. Choroidal bulging is a transient finding, as well, but localized, unique, and observed in the non-acute uveitic stage of the disease. It is probably related to posterior-segment active inflammation once it was observed concomitantly to other posterior inflammatory signs.

It is important to emphasize that simultaneous observation of EDI-OCT and ICGA shows transient dark dots at the exact location of the choroidal bulging. Actually, ICGA changes anticipated choroidal bulging detection on EDI-OCT in all cases. This truly does sound logical since bulging probably represents the accumulation of inflammatory elements/fluid associated to the presence of transient granulomas, which usually take some time to build up [10]. Indeed, choroidal bulging dynamism (appearance/disappearance) occurred in a faster way when compared to the distribution of dark dots on ICGA.

Therefore, the new EDI-OCT sign described here in the non-acute uveitic phase of the VKH disease does not substitute ICGA signs, but complement subclinical posterior segment inflammatory evaluation. Given that choroidal bulging has a dynamic nature, a histopathologic study would hardly be available. In addition, EDI-OCT is a non-invasive examination and can be easily performed when compared to ICGA, which is frequently unavailable in some centers. Further studies evaluating its specificity and sensitivity should be carried out.

In summary, we describe a novel EDI-OCT finding called ‘choroidal bulging’ in patients with VKH disease in the non-acute uveitic stage, who presented other clinical and/or angiographic sign of disease-related inflammation. We propose that this finding, which is easily and non-invasively identified through serial EDI-OCT evaluations, may help monitor posterior segment disease activity in patients with VKH disease in the non-acute uveitic stage.

## Competing interests

The authors declare that they have no competing interests.

## Authors’ contributions

VMS, FTS, CEH, and JHY participated in the acquisition, analysis, and interpretation of data. VMS, RAC, and JHY helped draft the manuscript. All authors (VMS, FTS, CEH, WYT, RAC, and JHY) revised it critically for important intellectual content and had approved the final manuscript.
